# Suggestions Relating to the Causes of Rapid Dental Decay

**Published:** 1882-03

**Authors:** C. W. Spalding

**Affiliations:** St. Louis.


### ARTICLE III.

### *Suggestions Relative to the Cause of Rapid Dental Decay.*

BY DR. C. W. SPALDING, OF ST. LOUIS.

[Read before Illinois State Dental Society.]

During the last year I have given considerable attention to the examination of the mixed fluids of the mouth which is commonly denominated saliva. This examination has been devoted chiefly to the chemical properties of this fluid, with a view of determining how far this property of saliva is responsible for that rapid decay of the teeth which practitioners of dentistry so often encounter.

In common, I believe, with many other practitioners, I have supposed that an acid condition of saliva prevails, or at least may usually be looked for in most cases of rapid decay of teeth. Under this impression I entered upon a series of chemical tests with a view of endeavoring to discover the cause of this assumed chemical condition of saliva.

My observations have resulted in the formation of an opinion amounting almost to conviction, that the agency heretofore ascribed to the chemical condition of saliva in promoting decay of teeth in healthy persons, has been largely over-estimated, if it has not been altogether erroneous.

My first experiments were made upon the saliva of a young girl (aged 12,) whose teeth were decaying rapidly, so much so that fillings previously inserted, that is, before the case came into my hands, whether of gold or of other materials, had lasted but a short time, and in whose teeth new cavities were constantly forming, and this at so rapid a rate that cavities of considerable size would form in the space of a few months, notwithstanding pretty thorough cleanliness and good general care. I looked to find an acid reaction of the saliva in this case, and had been reflecting on a course of medical treatment, having in view the correction of this supposed condition of the saliva. What, then, was my surprise, on finding after repeated tests, that the saliva of this young person exhibited in every test either a neutral or a slightly alkaline chemical reaction ! In no one of a large number of tests was there any, even the smallest acid reaction shown. I immediately sought other cases where a similar destructive process was going on, but the result in each was precisely the same as in the case just narrated. Indeed, in none was an acid condition present where the subject was in passably good health.

A professional friend also made similar tests in some very marked cases of rapid decay with the same results—no acid condition of saliva revealed.

I do not wish to be misunderstood. I am not combatting the idea that an acid condition of the saliva would promote decay of the teeth. I have no doubt it would do so, but I am endeavoring to show this condition of the saliva is not usual in states of ordinarily good health while the teeth do often rapidly decay, although the general health is not perceptibly, or at least not seriously, disturbed. The view I now present is, if the general health is good or fair, and the saliva normal, or nearly so, any rapid decay of the teeth must be attributed to some other cause than acid saliva. In certain localities favoring the lodgment and retention of food, decomposition of food substances probably takes place accompanied perhaps by the generation of acid. But many of the cavities of the class now under consideration, are located at points very unfavorable to retention, and it would not be fair to assume that decay occurring at these points was occasioned by products resulting from either the partial digestion, the oxidation, or any other change in food substances.

Are there any other known methods by which acids may be generated, to whose action this rapid decay of the teeth may be fairly attributed? Let the chemist answer. If yes, the methods should be sought out and explained that the chemical theory, as it relates to this class of cases, may be fairly presented. If, however, the chemical theory is found, on thorough investigation, to be inadequate to the production of this class of cavities, or of any cavities under the conditions I have presented, some other cause must be invoked. Are these undetermined causes external or internal to the teeth, or both internal and external? Under the suggestion that internal forces may become important predisposing causes to decay of the teeth, our thoughts turn at once to faulty development, and to the period when calcification of the teeth is going on. The calcification of the deciduous teeth commences about the fourth or fifth month of fetal life, and that of the permanent teeth during the ninth month of fetal life, or the first month after birth. It will therefore be seen that if medicinal treatment is to be attempted, it should be commenced at an early period of fetal growth. The calcific process continues uninterruptedly until the completion of the third molars, which it is probably safe to say does not take place earlier than the fifteenth year. Yet I think we may with fairness conclude that if the deciduous teeth are perfectly developed, no medication will be required to insure a similar condition of the permanent set.

If defective development is to be regarded as a primary predisposing cause of the early and speedy decay of the teeth, our attention should, first of all, be directed to the most effective means of correcting this evil. Evidently there is but one mode by which preventive or corrective methods can be more effectual previous to birth. It is only through the blood of the mother that ante-natal treatment can be successfully applied. But whether defective development is due to a scanty supply of nutrient material, or to a lack of assimilative or developmental force in the individual, is first to be considered, that treatment may conform to diagnosis. If to sufficient supply, would not the defect be manifest in the general osseous system? The amount of mineral salts requisite for the calcification of the teeth, —or I might limit the comparison to the enamel of the teeth,—is small, compared with that which is necessary to construct the entire skeleton. I may here notice the fact that bone and dentine are but imperfectly calcified at birth, and that deposition of lime salts goes on at a rapid rate for a considerable time after birth; and it should also be noted that the post-partum deposition of lime salts is probably slower and difficult in enamel than it is in either bone or dentine. This latter implies the greater need for ante-natal methods of insuring the perfect formation of enamel. But to return. The relatively small amount of lime salts composing the hard elements of an entire denture would scarcely be missed if the deficiency was equally distributed over the whole osseous system. If defective enamel is owing to an insufficiency of supply, would not the defect be general and show itself elsewhere as well as in the enamel of the teeth? I am aware that the tardy closure of the fontanelles, and the late hardening of the bones of the infant, are indications of a lack of ossific material; but these cases are exceptional, and include but a small proportion of those in which rapid decay of the teeth is a marked and prominent system. If this condition does not proceed from a deficiency of nutritive material, it must necessarily arise from a want of assimilative force. I am inclined to accept the latter hypothesis, and to attribute the faulty formation to what I may call defective lime function, rather than to a lack of the substance itself. If this view is correct, we are at once brought into the field of preventive medicine, a field that has been but imperfectly tilled, especially that branch which relates to medical treatment of the unborn child.

Probably Jenner was the first to bring into notice an useful example of preventive medicine when he announced the discovery that cow-pox inoculation was a preventive of small-pox. This was also one of the first revelations of the prophylactic utility of the law of similars. Grawvogl has more recently applied this law to the correction of hereditary ante-natal deformities, by the use of prophylactics administered to the mother. He reports a case of what may be called hereditary *hydrocephalus acutus*, although neither of the parents exhibited any tendency towards this disease. Two children had been born in this family and both had been attacked in early life, both cases terminating fatally. The parents are described as being healthy, both having blonde hair, thin skin and blue eyes, one being spare and the other of full habit. The disease began to be developed in both children coincidently with the eruption of the lower incisor teeth.

Osseons nutrition is always deficient in *hydrocephalus*, resulting probably from defective assimilation. This imperfection would probably manifest itself in the formation of the teeth as well as in the cranial bones, and, if so, the cause must have been in operation previous to their eruption. This later event is usually accompanied by considerable disturbance of the nutritive processes, and this disturbance may well be supposed to occasion manifestation of any predisposing causes of disease that had heretofore lain dormant. Hence, the concurrence of *hydrocephalus* and dentition.

In the treatment of the above named case sulphur and calcarea phos. were given, one dose of either on alternate days to the mother, commencing at the beginning of gestation and continuing to the birth of the child. Two additional children have been born of the same parents, both under this treatment of the mother, and both now respectively five and three years of age, are reported as being fine, healthy children, entirely free from any hydrocephalic symptoms. This case was not reported for the reason that it was either a solitary or an unusual one, but for the purpose of illustration only. Similar treatment has been pursued in other families where there had previously been a hydrocephalic child, and the same good results have uniformly followed. Hydrocephalic children have also been successfully treated by similar means, with the difference that sulphur was not indicated after the disease had made its appearance, and was, therefore, omitted.

Another interesting case of preventive medicine has been recently reported by J. C. Burnett, M. D., of London, England. In 1874, Dr. B. was consulted by a gentleman about his children, the youngest of whom had double harelip. The one next older had a defect in the upper lip, amounting almost to hare-lip, while the third or oldest child had no similar defect. The first born being perfect, the second having an insufficiency of the upper lip, and the third having double hare lip, it was thought, taking all the circumstances into consideration, that the fourth child would be likely to have double hare-lip and possibly cleft-palate. It was determined to endeavor to prevent so unfortunate an occurrence, and an attempt at ante-natal treatment was made with that view. Calcarea sulph. was chosen in this case in preference to calcarea phos. by reason of the psoric diathesis of the mother. At full term the mother gave birth to a perfect child, and subsequently the same treatment was followed through a second pregnancy with a like result.

The *Practitioner* for December, 1878, contains a paper "On the Preventive Treatment of Cleft-palate, Hare-lip, etc.," by Thomas P. Tuckey, M. D., of County Cork, Ireland. He reports the following case:

Mrs. H., aged thirty-five, mother of six children. Every one of the children has had hare-lip, two having also had cleft-palate. The mother could not call to mind any case of hare-lip in her own or husband's family. Both parents were strong, healthy persons, neither having ever been seriously ill. Dr. Tuckey proposed ante-natal treatment.

A pregnancy occurred when the doctor prescribed the following mixture:

℞. Calcis. phos. - - - ʒj. grs. 20.

Calcis. Carb. - - - ʒj.

Bicarb. magnes

Chlorid sodii

Sodæ Phosph. aa—|—ss. M.

The above to be added to an 8 oz. mixture composed of gelatine gum arbaic, syrup of ginger and cinnamon water; one dram, three times daily.

As clefts in the palate and lip are said to be due to arrest of development prior to the end of the third month, Mrs. H. was put upon this mixture at once. It was no doubt intended to represent the constituents of bone. The essential parts are the lime, magnesia and phosphorus.

"The woman took her medicine regularly until the fourth month; at her full time she was delivered of a girl without a trace of deformity about her lips or palate."

Dr. T. reports another case, Mrs. L. "mother of eight children, most of whom had cleft-palate and hare-lip; in four of them the hare-lip was double, and more shocking objects of deformity he had never seen. One boy was perfectly repulsive. The woman believed herself pregnant, and was at once put on the medicine. She went her full time, bore a girl without hare lip, indeed, but who evidently had had one in utero; for the lip, though united, was united crookedly, and one side was puckered up, as if by a slight and narrow burn."

It may be objected that these cases prove nothing; that there is no positive evidence that the same children would not have been born without deformity if no medicine had been given. To this I answer, I am only citing history, leaving every one free to draw his own inferences. Besides, I am only making suggestions for the purpose of drawing attention to the subject with a view to further investigation, in which I trust the members of this Society will join.

Embryology teaches us that the face is formed of a central portion produced from the frontal process, and of right and left lateral parts derived from the extremity of the first visceral arch. From this arch proceed the parts from which the superior and inferior maxillaries are subsequently developed. At the period in developmental growth when superior maxillary unites with the frontal process, a cavity remains which constitutes the nasal cavity. The palate and intermaxillary bones bridge this space, and thus the nose is separated from the mouth. There are many varieties of congenital cleft of the palate, differing, however, only in degree, while all of them may be said to result from arrested development. Whenever the inter-maxillary fails to unite with the maxillary bone, a cleft is the result. The same causes which produce the cleft, produce the corresponding hiatus in the soft palate or lip.

In conclusion I wish to say that it does not appear to me to be a legitimate inference that this arrest of development is due to a lack of nutritive material. The maxillaries and all other parts of the osseous structure complete themselves in the usual way. The arrest of development is limited to a specific and comparatively insignificant anatomical part. If the arrest was due to a lack of material, would it not be likely to include a larger area, and at least affect other parts that calcify coincidently with those now under consideration?

